# Profiling of Histone Modifications Reveals Epigenomic Dynamics During Abdominal Aortic Aneurysm Formation in Mouse Models

**DOI:** 10.3389/fcvm.2020.595011

**Published:** 2020-10-30

**Authors:** Jacob Greenway, Nicole Gilreath, Sagar Patel, Tetsuo Horimatsu, Mary Moses, David Kim, Lauren Reid, Mourad Ogbi, Yang Shi, Xin-Yun Lu, Mrinal Shukla, Richard Lee, Yuqing Huo, Lufei Young, Ha Won Kim, Neal L. Weintraub

**Affiliations:** ^1^Departments of Medicine, College of Nursing at Augusta University, Augusta, GA, United States; ^2^Vascular Biology Center, Medical College of Georgia, College of Nursing at Augusta University, Augusta, GA, United States; ^3^Department of Population Health Sciences, College of Nursing at Augusta University, Augusta, GA, United States; ^4^Department of Neuroscience and Regenerative Medicine, College of Nursing at Augusta University, Augusta, GA, United States; ^5^Department of Surgery, College of Nursing at Augusta University, Augusta, GA, United States; ^6^Department of Cell Biology and Anatomy, College of Nursing at Augusta University, Augusta, GA, United States; ^7^Department of Physiological and Technological Nursing, College of Nursing at Augusta University, Augusta, GA, United States

**Keywords:** abdominal aortic aneurysm, angiotensin II, calcium chloride, histone modification, methylation, acetylation

## Abstract

**Introduction:** Abdominal aortic aneurysms (AAA) are characterized by localized inflammation, extracellular matrix degradation, and apoptosis of smooth muscle cells, which together lead to progressive and irreversible aortic dilation. Major risk factors for AAA include smoking and aging, both of which prominently alter gene expression via epigenetic mechanisms, including histone methylation (me) and acetylation (ac).However, little is known about epigenomic dynamics during AAA formation. Here, we profiled histone modification patterns in aortic tissues during AAA formation in two distinct mouse models; (1) angiotensin II (AngII) infusion in low density lipoprotein receptor (LDLR) knockout (KO) mice, and (2) calcium chloride (CaCl_2_) application in wild type mice.

**Methods and Results:** AAA formed in both models, in conjunction with enhanced macrophage infiltration, elastin degradation and matrix metalloproteinases expression as evaluated by immunohistochemistry. To investigate the histone modification patterns during AAA formation, total histone proteins were extracted from AAA tissues, and histone H3 modifications were quantified using profiling kits. Intriguingly, we observed dynamic changes in histone H3 modifications of lysine (K) residues at different time points during AAA formation. In mature aneurysmal tissues at 3 weeks after AngII infusion, we detected reduced K4/K27/K36 monomethylation, K9 trimethylation K9, and K9/K56 acetylation (<70%), and increased K4 trimethylation (>130%). Conversely, in CaCl_2_-induced AAA, K4/K9/K27/K36/K79 monomethylation and K9/K18/K56 acetylation were reduced in AAA tissues, whereas K27 di-/tri-methylation and K14 acetylation were upregulated. Interestingly, K4/K27/K36 monomethylation, K9 trimethylation, and K9/K56 acetylation were commonly downregulated in both animal models, while no H3 modifications were uniformly upregulated. Western blot of AAA tissues confirmed markedly reduced levels of key H3 modifications, including H3K4me1, H3K9me3, and H3K56ac. Furthermore, pathway enrichment analysis using an integrative bioinformatics approach identified specific molecular pathways, including endocytosis, exon guidance and focal adhesion signaling, that may potentially be linked to these histone H3 modifications during AAA formation.

**Conclusions:** Dynamic modifications of histone H3 occur during AAA formation in both animal models. We identified 6 discreet H3 modifications that are consistently downregulated in both models, suggesting a possible role in AAA pathobiology. Identifying the functional mechanisms may facilitate development of novel strategies for AAA prevention or treatment.

## Introduction

Abdominal aortic aneurysms (AAA) are a relatively common cause of death and are frequently asymptomatic until rupture occurs (1, 2). AAA repair is associated with significant morbidity and mortality, particularly when performed emergently; thus far, no medical therapy has proven effective in preventing AAA growth or rupture (3). Human AAA are typically characterized by elastin degradation, loss of vascular smooth muscle cells (VSMCs), and immune cell infiltration (4). Smoking, age, male sex, hypertension, and hypercholesterolemia are important risk factors for AAA, but the underlying mechanisms that lead to AAA formation are incompletely defined.

Epigenetic regulation refers to potentially stable and heritable changes in gene expression that arise independent of alterations in the primary DNA sequences. Epigenetic modifications occur dynamically in response to various environmental stimuli, and recent studies have shown that several risk factors for AAA, including smoking and aging, can dysregulate gene expression through epigenetic mechanisms (5, 6). Major processes associated with epigenetic control of gene expression include DNA methylation, histone modifications and non-coding RNA (7). Among these epigenetic mechanisms, histone (H) modifications fundamentally change chromatin structure and gene transcription, thereby regulating key cellular mechanisms and functions. Histone methylation is a post-translational process whereby methyl groups are attached to H3 and H4 proteins by enzyme histone methyltransferases (HMTs), thus altering the histone's interactions with DNA and nuclear proteins. Either transcriptional repression or activation can occur, depending on the site of methylation and its impact on chromatin structure. Histone acetylation refers to the process whereby acetyl groups are added to histone proteins, specifically to the lysine residues, by histone acetyltransferase (HAT). Histone acetylation typically leads to a transcriptionally active chromatin structure. Separate groups of enzymes, termed histone demethylases and deacetylases, remove the methyl and acetyl groups, respectively, thus allowing for dynamic regulation of gene expression. Epigenetic mechanisms are associated with many human diseases, including cardiovascular disease, cancer and metabolic disease (8–10). However, epigenetic mechanisms associated with AAA have not been extensively investigated, and most available data have been inferred from studies of other types of vascular disease or pathology. Hence, understanding the epigenetic mechanisms that are operative in AAA could potentially lead to development of novel therapeutics that ameliorate AAA growth and rupture.

Several animal models have been developed to study AAA formation. Angiotensin II (AngII) infusion by osmotic minipump and periaortic calcium chloride (CaCl_2_) application are well-established animal models of AAA. The presentation of AAA in these models is not identical to the human disease, but they serve as valuable models that share key features of human AAA (11). The AngII model is characterized by elastic degradation, macrophage infiltration, thrombus formation, aortic dissection, and rupture. However, these mice typically develop aneurysms in the suprarenal aorta, as opposed to the infrarenal aorta in humans. In addition, aortic rupture is an early event in the AngII-infusion model, associated with medial dissection, while in human AAA, rupture is most often a late event associated with pronounced dilation. On the other hand, the CaCl_2_ application model induces markedly dilated AAA in the infrarenal location. CaCl_2_-induced AAA also exhibit many pathological characteristics observed in human AAA, including calcification, profound neutrophil infiltration and elastin degradation, neovascularization, and VSMC apoptosis. However, the aneurysms do not rupture or develop thrombus formation, nor are they associated with atherosclerosis, which are typical features of human AAA (11).

Here, using two distinct animal models of AAA, we focused on profiling aortic histone H3 modifications during AAA formation. We detected dynamic histone modification patterns occurring in a time- and model-dependent fashion, and we identified several H3 modifications that were highly conserved between the two models. Furthermore, pathway enrichment analysis using an integrative bioinformatics approach identified specific molecular signaling pathways that may potentially be linked to these histone H3 modifications during AAA formation. Our findings set the stage for future investigations into the role of these dynamic histone H3 modifications in AAA pathogenesis.

## Materials and Methods

### AngII-Induced AAA Model

Animal experimental protocols were approved by the Institutional Animal Care and Use Committee at the Medical College of Georgia at Augusta University and complied with National Institute of Health guidelines. Low density lipoprotein receptor (LDLR) knockout (KO) mice were purchased from Jackson Laboratory. Ang II (1,000 ng/kg/min, Enzo Life Sciences) was infused into male LDLR KO mice via osmotic minipumps (ALZET Model 2004) as previously reported (12, 13). One to three weeks after minipump implantation, mice were euthanized and aortic outer diameter was measured. Aortic tissues were harvested for immunohistochemical analysis, histone H3 analysis and Western blot.

### CaCl_2_-Induced AAA Model

Male C57Bl/6 mice (Jackson Laboratory) were treated with CaCl_2_ (0.5 mol/L, Sigma-Aldrich) for AAA induction as described previously (12). After 1–3 weeks, animals were anesthetized, aortic outer diameter was measured, and aortas were harvested for further analysis.

### Aortic Tissue Collection

Aortic tissue samples were harvested as described previously (12). Briefly, after blood was withdrawn from the right ventricle, aortas were irrigated with cold PBS through the left ventricle, and the peri-adventitial tissue was dissected carefully from the wall of the aorta. The abdominal aorta, from the last intercostal artery to the ileal bifurcation, was sectioned and the aneurysmal areas were fixed in paraformaldehyde (4% wt/vol) for immunohistochemistry, or processed for histone extraction as described below.

### Immunohistochemistry

Paraffin-embedded cross sections of aortas were used for hematoxylin and eosin (H&E), Verhoeff van Gieson (VVG) and immunostaining staining for Mac-3, matrix metalloproteinase (MMP)-2 and−9. Antibodies for Mac-3 (BD Pharmingen) and MMP-2/-9 (Calbiochem) were used with the HistoMouse-SP kit (Invitrogen) or DAB Substrate kit (Vector Labs).

### Histone Extraction and Quantification of Histone Modifications

Total histone proteins were extracted from aortas using EpiQuik™ Total Histone Extraction Kit (Epigentek). Major histone H3 modifications were quantified using EpiQuik™ Histone H3 Modification Multiplex Assay Kit (Epigentek). Pooled aortic histone extracts from three mice were subjected to the assay and 100 ng of total histone proteins were used.

### Western Blotting

Protein extraction and western blotting were performed as described previously (12). H3K4me1, H3K9me3, H3K56ac and total histone H3 antibodies were purchased from Cell Signaling Technology. Pooled aortic histone extracts from three mice were subjected to the assay.

### Quantitative PCR

Total RNA was extracted from whole aortas using QIAzol lysis reagent and purified with the RNeasy lipid tissue mini kit (Qiagen) per manufacturer's protocol. Real-time quantification of mRNA was performed using Power SYBR® Green RNA-to-CT™ 1-Step Kit (Applied Biosystems). Normalized Ct values were subjected to statistical analysis, and the fold difference was calculated by the ΔΔCt method.

### Bioinformatic Analysis

Chromatin immunoprecipitation followed by sequencing (ChIP-Seq) data for mouse heart and human aorta were downloaded from the website of the Encyclopedia of DNA Elements (ENCODE) project (14). The R package ChIPseeker (15) was used to retrieve the nearest genes around the peak and to annotate the genomic region of the peak, while the UCSC build hg19 and mm10 annotations were used to annotate the known genes for human and mouse data, respectively. The transcription start site (TSS) region of each gene was defined from −3kb to +3kb. Next, the annotated genes were used as an input for functional enrichment analysis, which was performed using the Gene Ontology (GO) terms, Kyoto Encyclopedia of Genes and Genomes (KEGG) pathways and Reactome pathways databases, respectively.

### Statistical Analysis

All statistical analysis was performed using Graphpad Software and results are expressed as mean ± SEM. Differences between two groups were analyzed by Student's *t*-test. *P*-values <0.05 were considered to be significant.

## Results

### AAA Formation by AngII Infusion and Periaortic CaCl_2_ Application in Mice

**Figure 1:**
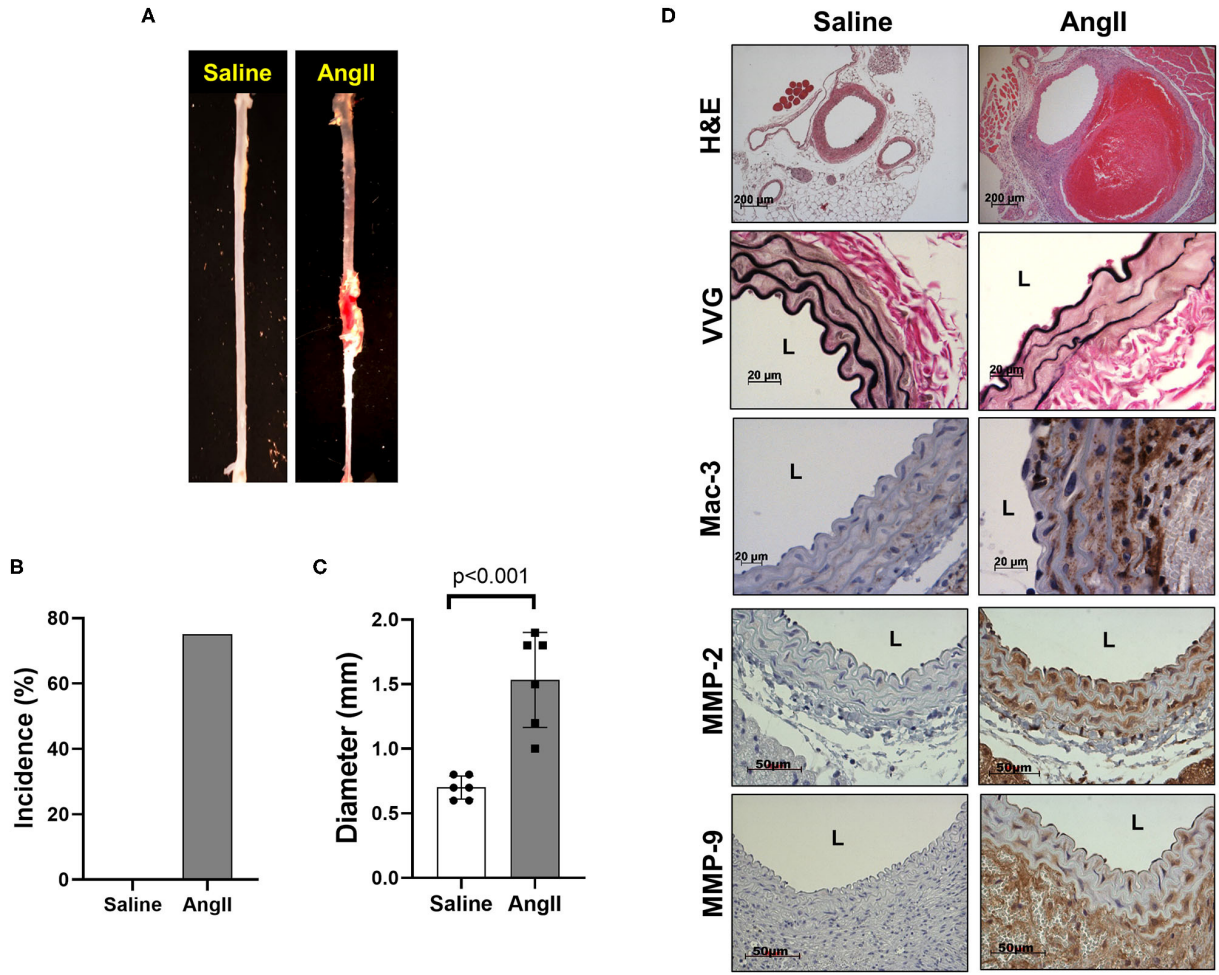
AngII formation by AngII infusion. AngII was infused via osmotic minipump in LDLR KO mice for up to 3 weeks. **(A)** Representative image of AAA formation by AngII infusion in the suprarenal area of mouse aorta. **(B)** AAA incidence. **(C)** Aortic diameter. **(D)** Representative histology; H&E, VVG (elastin fragmentation), Mac-3 (macrophages) and MMP-2/-9, L, lumen.

To induce AAA in mice, we employed two distinct animal models, AngII infusion and CaCl_2_ application. Phenotypic differences of AAA between two animal models are summarized in the Table 1. As shown in Figure 1, AngII infusion for 3 weeks induced AAA in nearly 80% of the LDLR KO mice, resulting in a significant increase in suprarenal aortic diameter as visualized macroscopically and confirmed by H&E staining (Figures 1A–D). Elastin degradation, MMP expression and vascular inflammation were markedly increased in aortas of mice infused with AngII as compared to control (saline infusion) as evaluated by VVG staining, MMP-2/-9 staining, and MAC-3 staining (Figure 1D).

**Table 1:** Phenotypic differences of AAA between two animal models.

**Human AAA features**	**AngII model**	**CaCl_**2**_ model**
Medial degeneration	X	X
Inflammatory cell infiltration	X	X
VSMC apoptosis	X	X
Oxidative stress	X	X
Thrombus formation	X	
Aortic rupture	X	
Neutrophil infiltration		X
Anatomic location (infrarenal)		X
Risk factors: hypertension, atherosclerosis, male predisposition	X X X	

**Figure 2:**
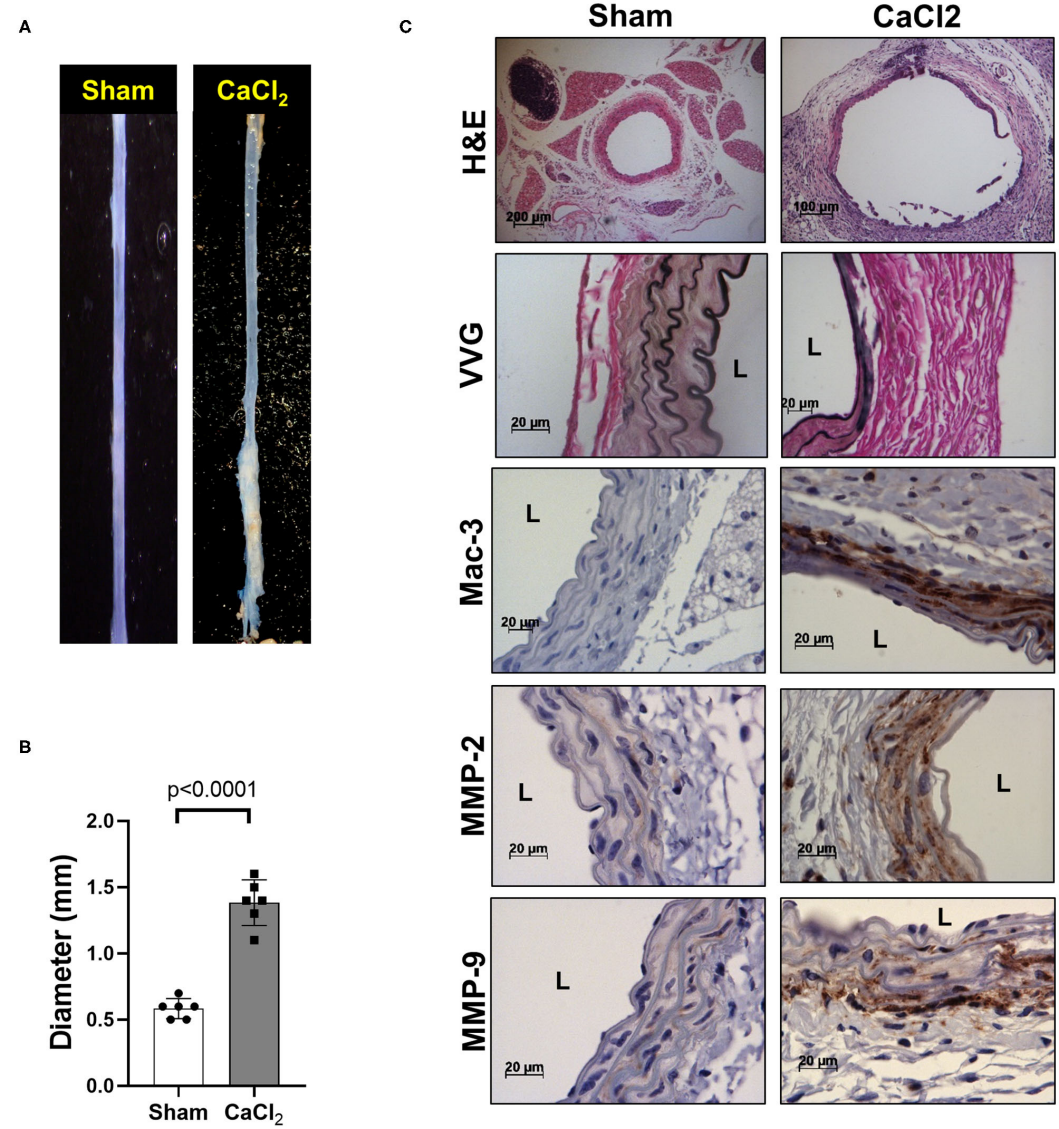
AAA formation by periaortic CaCl_2_ application. CaCl_2_ was applied to the infrarenal aorta of C57Bl/6 mice, which were sacrificed at 3 weeks after CaCl_2_ application. **(A)** Representative image of AAA formation by CaCl_2_ application in infrarenal area of mouse aorta. **(B)** Aortic diameter. **(C)** Representative histology; H&E, VVG (elastin fragmentation), Mac-3 (macrophages) and MMP-2/-9, L, lumen.

We also confirmed that infrarenal aortic diameter was significantly increased by periaortic CaCl_2_ application in wild-type (C57Bl/6) mice after 3 weeks (Figures 2A,B), in conjunction with severe elastin degradation (VVG staining), increased macrophage infiltration (Mac-3 staining) and MMP-2/-9 expression (MMP-2/-9 staining, Figure 2C).

### Profiling of Histone H3 Modifications in AngII-Induced AAA

**Figure 3:**
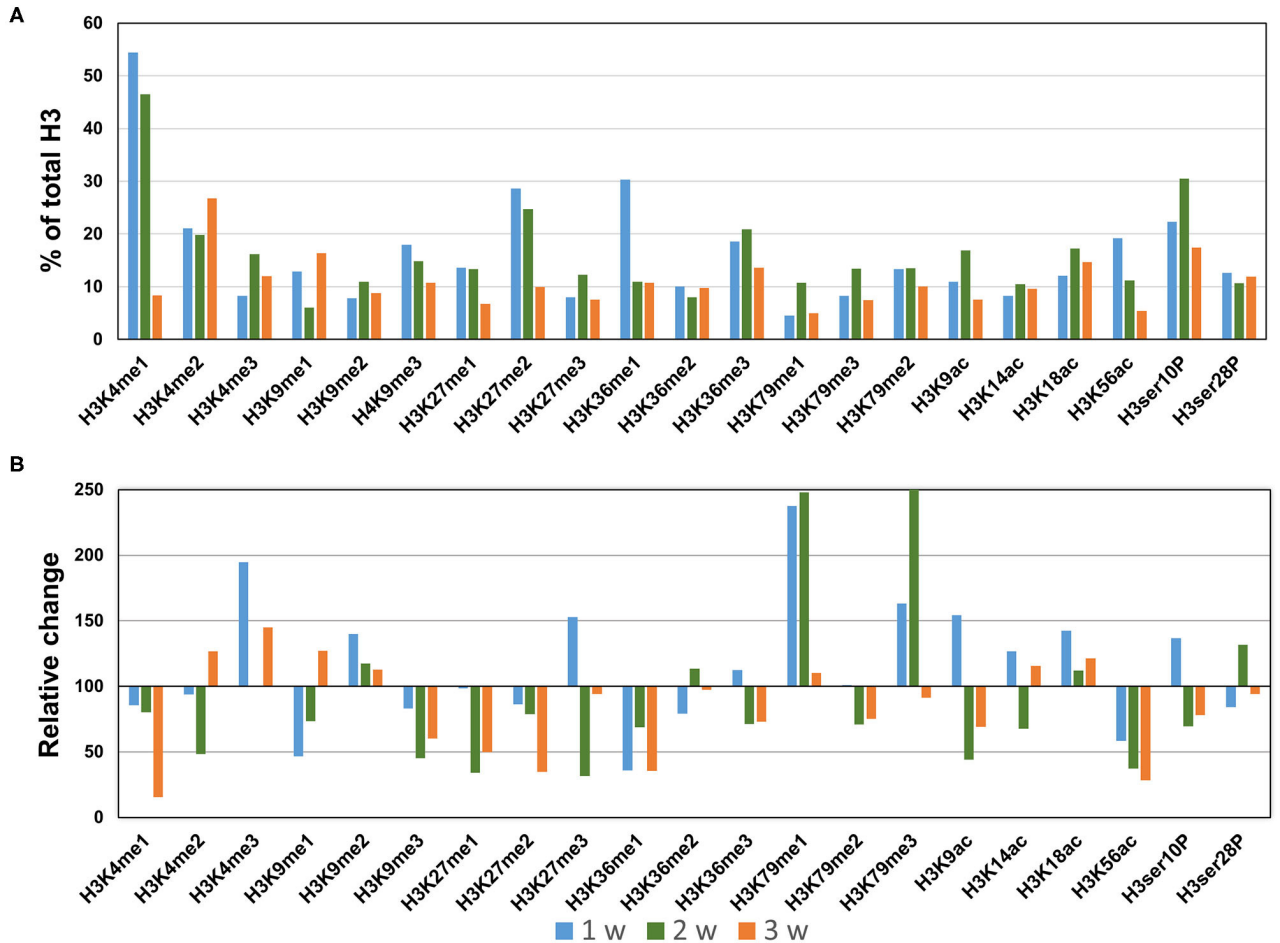
Profiling of major histone H3 modifications in AngII-induced AAA tissues. Total histone proteins were extracted from AAA tissues (at 1, 2, 3 weeks, respectively), and histone H3 modifications were quantified using profiling kits. Pooled aortic histone extracts from three mice were subjected to the assay. **(A)** Percentage of total histone H3. **(B)** Relative changes of histone H3 modifications.

In the AngII infusion model, we identified a unique H3 modification profile at each time point (1, 2, and 3 weeks after AngII infusion). At 1 week after AngII infusion, when inflammation and oxidative stress are prevalent and preceed the formation of AAA, we observed significant upregulation (>130%) of methylation of K4, K9, K27, K79 (H3K4me3, H3K9me2, H3K27me3, H3K79me1/me3), acetylation of K9, K18 (H3K9ac, H3K18ac) and phosphorylation of ser10 (H3ser10P), while there was significant downregulation (<70%) of H3K9me1, H3K36me1, and H3K56ac (Figures 3A,B). At 2 weeks after AngII infusion, which represents the middle stage of AAA formation, mono- and try-methylation of K79 (H3K79me1/me3) and phosphorylation of ser28 (H3ser28P) was upregulated, whereas methylation (H3K4me2, H3K9me3, H3K36me1, H3K27me1, H3K27me3, H3K36me1) and acetylation (H3K9ac, H3ser10P, H3K9ac, H3K14ac, H3K56ac) were largely downregulated (Figures 3A,B). At 3 weeks after AngII infusion, at which time the AAA is mature, only H3K4me3 was upregulated, while H3K27me2, H3K4me1, H3K9me3, H3K27me1, H3K36me1, H3K9ac, and H3K56ac were downregulated (Figures 3A,B). These results demonstrate that dynamic H3 modifications occur during AAA formation induced by AngII.

### Profiling of Histone H3 Modifications in CaCl_2_-Induced AAA

**Figure 4:**
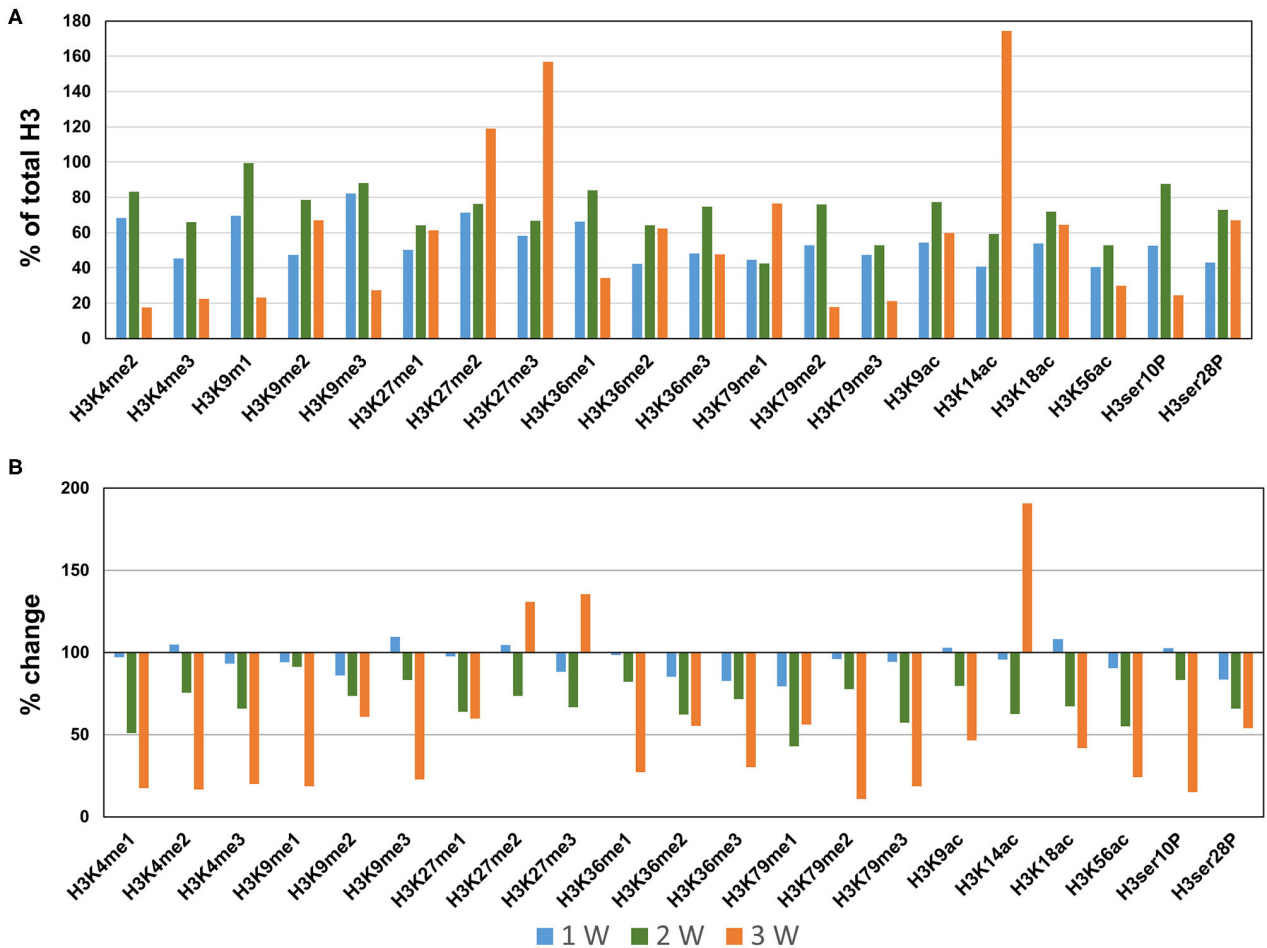
Profiling of major histone H3 modifications in CaCl_2_-induced AAA tissues. Total histone proteins were extracted from AAA tissues (at 1, 2, 3 weeks, respectively), and histone H3 modifications were quantified using profiling kits. Pooled aortic histone extracts from three mice were subjected to the assay. **(A)** Percentage of total histone H3. **(B)** Relative changes of histone H3 modifications.

Next, we profiled the histone H3 modifications in CaCl_2_-induced AAA. In contrast to the AngII-induced AAA model, H3 modifications were neither significantly upregulated nor downregulated at 1 week after CaCl_2_ application (Figures 4A,B). At 2 weeks after CaCl_2_ application, downregulation of K4/K9/K27/K36/K79 monomethylation, K9/K18/K56 acetylation, and H3ser28P phosphorylation was detected (Figures 4A,B). Additional histone modifications (H3K4me1/2/3, H3K9me1/2/3, H3K27me1, H3K36me1/2/3, H3K79me1/2/3, H3K9ac, H3K18ac, H3K56ac, H3ser10P, H3ser28P) were observed to be downregulated 3 weeks after CaCl_2_ application (Figures 4A,B). On the other hand, only H3K27me2/3 and H3K14ac were found to be upregulated at this latter time point.

### Comparing H3 Modifications Between AngII- and CaCl_2_-Induced AAA

**Figure 5:**
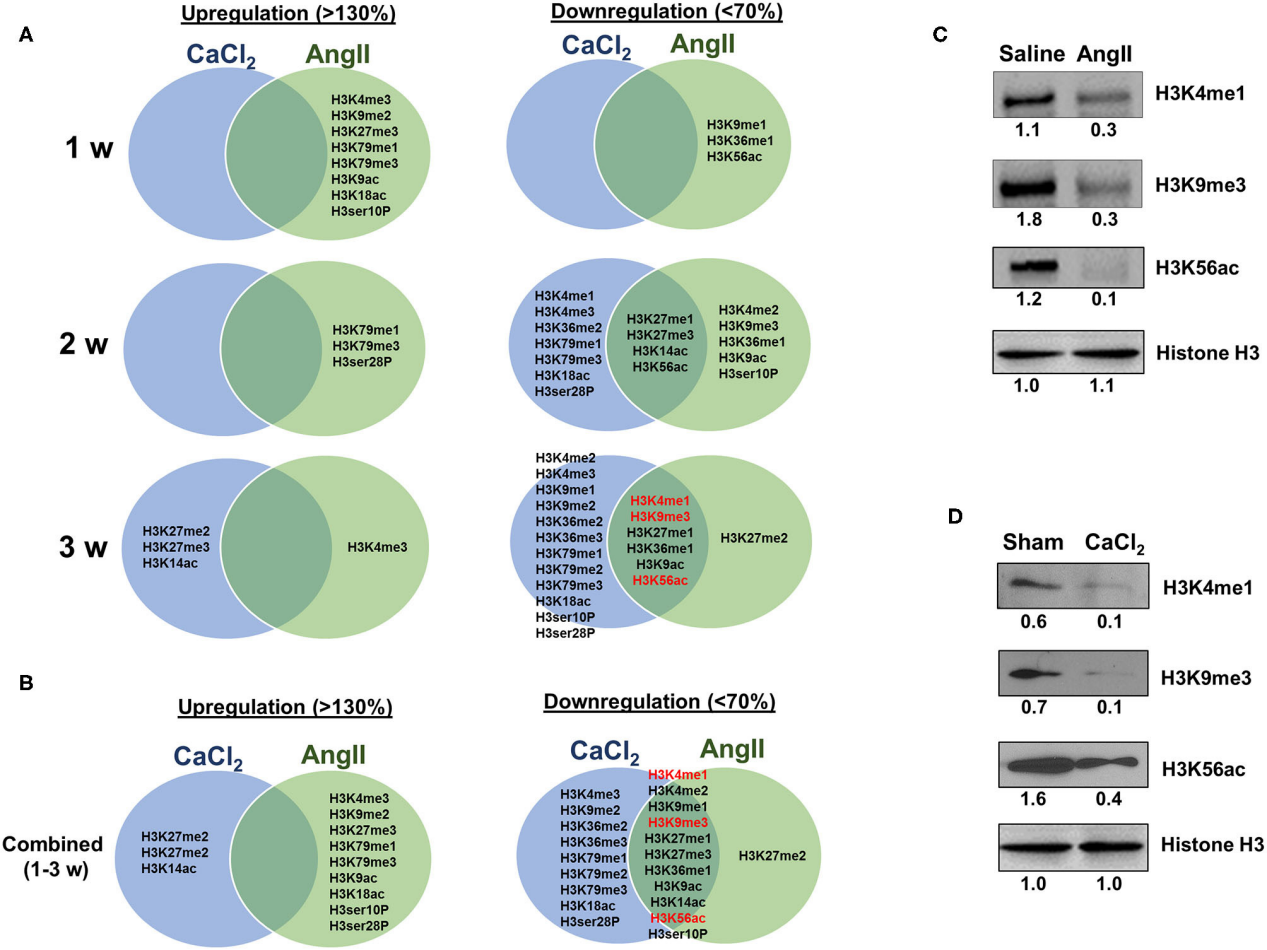
Comparing H3 modifications between AngII- and CaCl_2_-induced AAA. **(A)** Summary of upregulated (>130%) and downregulated (<70%) histone H3 modifications in AngII- and CaCl_2_-induced AAA tissues in each time point (1, 2, 3 weeks, respectively). Venn diagram was used to demonstrate the overlapping histone H3 modifications between the two animal models of AAA. **(B)** Combined data at all time points (1–3 weeks). Key overlapping H3 modifications (H3K4me1, H3K9me3, and H3K56ac) in AngII-induced **(C)** and CaCl_2_-induced AAA **(D)** were verified by Western blot. Pooled aortic histone extracts from three mice were subjected to Western blot. Number represents the quantitative expression levels, normalized by histone H3 in saline or sham group.

As described above, the two animal models we employed exhibit both overlapping and contrasting pathophysiological features, which presumably result from common and distinct biochemical and molecular mechanisms, respectively. Thus, we sought to identify overlapping histone H3 modifications observed in both models. Interestingly, we found that the overlapping H3 modifications between the models were all downregulated (Figures 5A,B). Four modifications (H3K27me1/me3, H3K14ac, and H3K56ac) were found to be commonly downregulated 2 weeks after AAA induction, and six discreet modifications (H3K4me1, H3K9me3, H3K27me1, H3K36me1, H3K9ac, and H3K56ac) were uniformly downregulated at 3 weeks (Figure 5B). Western blot was performed to verify key H3 modifications (H3K4me1, H3K9me3, and H3K56ac) consistently downregulated in both models at 3 weeks after AAA induction (Figures 5C,D).

### Functional Enrichment Analysis of Genes With H3 Modifications

**Figure 6:**
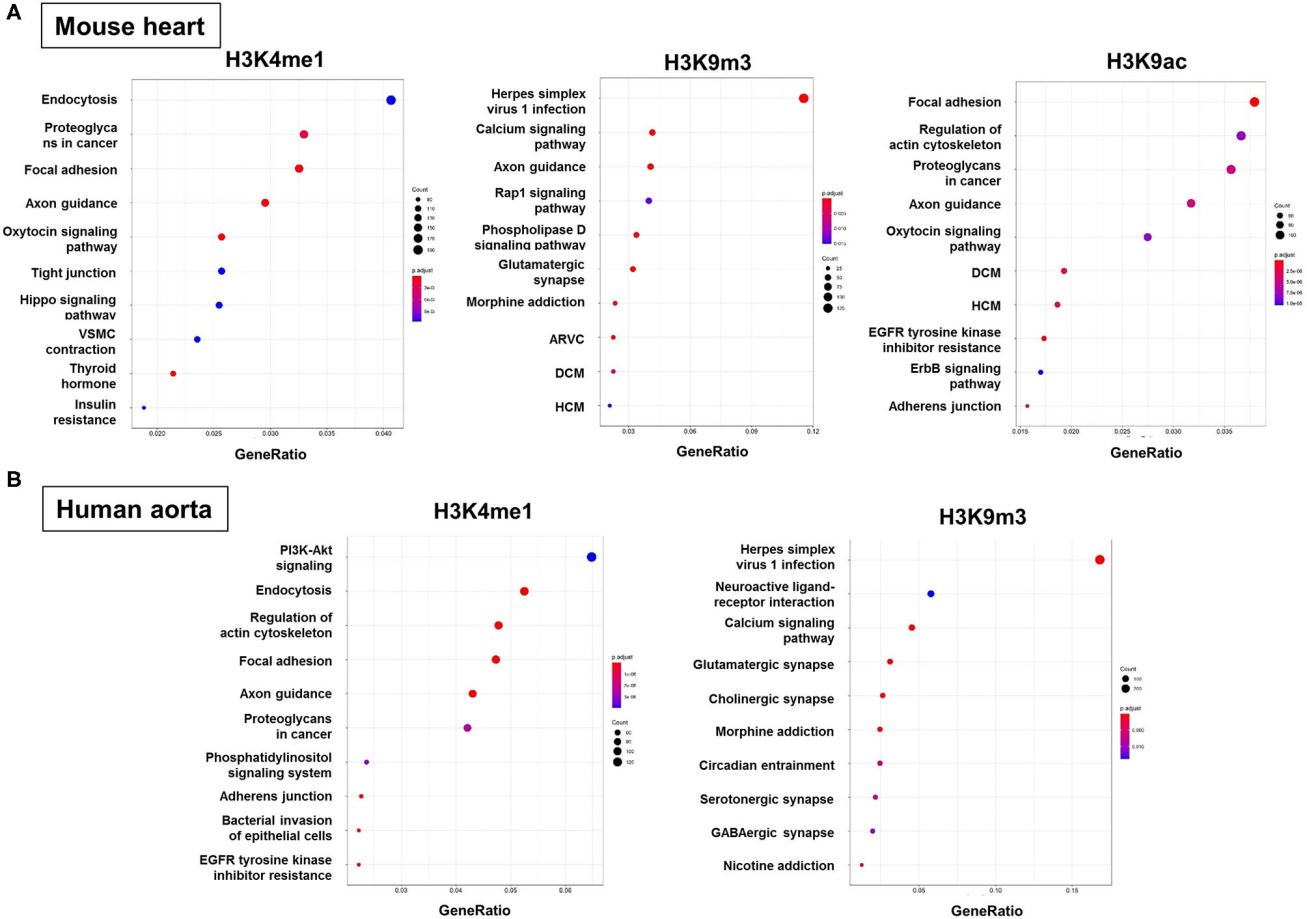
Functional enrichment analysis of genes with H3 modifications. GO terms, KEGG pathways and Reactome pathways databases were used as an input for functional enrichment analysis. Pathways and GO terms that are enriched for genes with H3K4me1, H3K9m3, and H3K9ac in mouse heart tissue **(A)** and genes with H3K4me1 and H3K9m3 in human aorta tissue **(B)**. The results of GO terms and Reactome pathways are shown in Supplemental Materials. GO, gene ontology; KEGG, kyoto encyclopedia of genes and genomes; ARVC, arrhythmogenic right ventricular cardiomyopathy; DCM, dilated cardiomyopathy; HCM, hypertrophic cardiomyopathy.

To predict the molecular mechanisms and biological functions of H3 modifications in AAA pathogenesis, we performed pathways and GO terms enrichment analyses using publicly available ChIP-Seq data from the ENCODE project. Since no Chip-seq data from mouse aorta are available, we examined the pathways and GO terms that are enriched in genes with H3K4me1, H3K9m3, and H3K9ac in mouse heart tissue and genes with H3K4me1 and H3K9m3 in human aorta tissue (Figure 6 and Supplementary Figures 1–7). Interestingly, some pathways and GO terms were found to be common in both mouse and human tissues. For instance, the KEGG pathways such as endocytosis, axon guidance, regulation of actin cytoskeleton and focal adhesion were significantly enriched of genes with H3K4me1, while pathways such as calcium signaling, glutamatergic synapse and Herpes simplex virus 1 infection were significantly enriched of genes with H3K9m3 (Figure 6). In support of our bioinformatics data, we measured mRNA expression of several genes which were randomly selected from the proposed signaling pathways. Interestingly, we found that gene expression of smooth muscle alpha-2 actin (*Acta2*), which is associated with actin cytoskeleton regulation, was significantly reduced in AngII-induced AAA tissues as compared to saline control. Furthermore, expression of integrin-linked kinase (*Ilk*), a focal adhesion and cytoskeleton-associated molecule, showed a trend toward reduced expression in AAA tissues (Supplementary Figure 8).

## Discussion

AAA pathogenesis is influenced by environmental, genetic and epigenetic regulatory mechanisms. Two of the major risk factors for AAA, smoking and age, prominently alter gene expression via epigenetic mechanisms, including histone methylation and acetylation (16–18). However, little is known regarding the epigenomics of AAA formation. In this study, using two dinstinct animal models of AAA, we profiled histone H3 modifications and analyzed their dynamic changes during AAA formation. Moreover, we identified those histone H3 modifications which overlapped in the two animal models of AAA. Interestingly, we detected consistent downregulation of H3K4me1, H3K9me3, H3K56ac levels in both animal models, while no H3 modifications were found to be consistently upregulated. Furthermore, pathway enrichment analysis of genes by integrative bioinformatics approach suggested that specific functional pathways, including endocytosis, exon guidance and focal adhesion signaling, may be associated with these overlapping histone H3 modifications during AAA formation.

Histone modifications are known to be key epigenetic mechanisms associated with vascular diseases such as atherosclerosis. However, the association of histone modifications with AAA pathogenesis is not well defined. A previous study showed that global acetylation rates of H3, and specific acetylation at H3K9, were lower in regulatory T cells of patients with established AAA, which was speculated to be associated with reduced T cell numbers and transcriptional activity (19). These results are consistent with our data from animal models in that acetylation at H3K9 was also decreased in mature AAA tissues. In contrast, Han et al. detected increased expression of histone acetyltransferase enzymes, along with increased histone acetylation modifications, in human AAA tissues obtained during elective surgical repair as compared to healthy control aorta obtained from organ donors suffering traumatic injury (20). Moreover, Gomez et al. showed that H3K9 acetylation and H3K4 methylation were increased at the Smad2 promoter of VSMCs derived from thoracic aortic aneurysm tissues as compared to control aorta from organ transplant donors (21). These human studies are not strictly comparable to our animal study, with principal differences not only related to species, but also to disease chronicity, associated medical conditions, predisposing risk factors, control tissues, etc. Further in-depth studies are required to resolve these differences and define the processes that regulate histone methylation and acetylation patterns in AAA.

Acetylation of H3K4 and H3K9 is in part regulated by silent mating type information regulation 2 homolog 1 (Sirt1), an NAD^+^-dependent class III histone deacetylase that plays an important role in genome stability through deacetylation of N-terminus tails of acetylated histones. We recently reported that Sirt1 activity is significantly reduced in AAA tissues and is mechanistically linked to AAA pathogenesis (22). Interestingly, Sirt1 was also reported to regulate histone methyltransferase SUV39H1-dependent H3K9me3 (23), levels of which are reduced by smoking and aging, key risk factors for AAA (24, 25). Our observation that H3K9me3 is one of the most downregulated H3 modifications in AAA potentially implicates dysregulation of the Sirt1/SUV39H1/H3K9me3 axis as a mechanism involved in AAA pathogenesis, which warrants further investigation.

In this study, we performed functional enrichment analysis of genes associated with histone H3 modifications to predict the cellular pathways involved in AAA pathogenesis. Since mouse aorta ChIP-seq databases do not exist, we probed databases of human aorta and mouse heart. Using the human aorta database, we identified putative signaling pathways that could be potentially regulated by H3 modifications during AAA formation (e.g., PI3K-Akt pathway, endocytosis, actin cytoskeleton, focal adhesion for H3K4me1 and calcium signaling, glutamatergic synapse, cholinergic synapse, associated with H3K9me3). Interestingly, some pathways and GO terms were found to be common in both mouse heart and human aorta. (e.g., endocytosis, focal adhesion and exon guidance pathways, associated with H3K4me1). Previously, genome-wide association studies (GWAS) carried out by an international consortia on large sample sets of AAA cases has identified low density lipoprotein receptor-related protein 1 (LRP1) as one of the most significantly associated genes for AAA (26). Interestingly, LRP1 is known to be associated with VSMC and macrophage endocytosis, both of which were also predicted to be involved in AAA formation by our functional enrichment analysis (27). Our bioinformatic data also indicate that focal adhesion pathway may be involved in AAA; a previous study likewise reported that focal adhesion kinase, a cytoplasmic tyrosine kinase, plays an important role in the progression of aortic aneurysm by modulating macrophage behavior (28). Our data also predicted that actin cytoskeleton regulation may be a signaling pathway associated with AAA, and in keeping with this notion, we showed that expression of Acta2, a gene involved in actin cytoskeleton regulation, was significantly reduced in AAA tissues. Interestingly, Acta2 was previously reported to contribute to AngII-induced thoracic aortic aneurysms (TAA) and dissections (29). Moreover, mutations in Acta2 are associated with TAA in humans (30). We also detected a non-significant trend toward reduced expression of Ilk, a focal adhesion and actin cytoskeleton-associated gene, in AAA tissues. A previous study demonstrated that VSMC-specific deletion of Ilk increased TAA formation (31). Taken together, these findings suggest that Acta2 and Ilk can regulate not only TAA, but also AAA formation. Collectively, the data suggest that these key signaling pathways associated with histone H3 modifications may be linked to epigenetic regulatory mechanisms involved in AAA pathogenesis. However, these bioinformatic data need to be further verified experimentally in future studies.

Mechanisms of AAA formation are complex, and distinct cell types and molecular pathways are associated with different stages of AAA pathogenesis (e.g., initiation vs. progression). For example, extracellular matrix degradation is observed early in the initiation of AAA, while intraluminal thrombus formation and angiogenesis occur primarily in the progression and maturation stages. Although immune cell recruitment can be observed in all stages, innate immune responses, mainly mediated by neutrophils, are an initiating mechanism of AAA. Our results demonstrate that certain histone modifications are upregulated in the initiation stage, but downregulated in the maturation stage (e.g., H3K79me1/me3). These temporal changes in histone modifications may reflect the distinct cell types and molecular pathways involved at different stages of AAA formation, a hypothesis which will need to be addressed in future studies.

In conclusion, despite the limitations of this exploratory study, our results demonstrate the dynamic changes of histone H3 modifications occurring duing AAA formation and suggest underlying mechanisms that could serve as novel targets for AAA treatment. In future studies, ChIP-seq will be required to gain more insight into mechanisms of epigenetic regulation of AAA. Moreover, future studies are also required to identify specific cell types which are responsible for individual histone modifications detected in the whole aorta, which consists of numerous cell types, including VSMCs, endothelial cells, fibroblasts, immune cells, etc., which together orchestrate AAA formation. Finally, further investigations into histone H4 modifications and histone-modifying enzymes are required to fully define the epigenetic landscape associated with AAA formation.

## Data Availability Statement

The raw data supporting the conclusions of this article will be made available by the authors, without undue reservation.

## Ethics Statement

The animal study was reviewed and approved by Institutional Animal Care and Use Committee at the Medical College of Georgia at Augusta University.

## Author Contributions

HK and NW developed the conception and design of the study. JG, NG, SP, TH, MM, DK, LR, MO, and HK performed experiments and collected data. JG, YS, X-YL, MS, RL, YH, LY, HK, and NW analyzed, interpreted, and discussed data. JG, YS, HK, and NW wrote the manuscript. All authors contributed to the article and approved the submitted version.

## Conflict of Interest

The authors declare that the research was conducted in the absence of any commercial or financial relationships that could be construed as a potential conflict of interest.

## Abbreviations

AAA: Abdominal aortic aneurysms
ac: acetylation
Acta2: smooth muscle alpha-2 actin
AngII: Angiotensin II
CaCl_2_: calcium chloride
Chip-Seq: chromatin immunoprecipitation followed by sequencing
GO: Gene Ontology
H&E: hematoxylin and eosin
Ilk: intergrin-linked kinase
K: lysine
KEGG: Kyoto Encyclopedia of Genes and Genomes
KO: knockout
LDLR: low density lipoprotein receptor
LRP1: identified low density lipoprotein receptor-related protein 1
me: methylation
MMP: matrix metalloproteinase
P: phosphorylation
Sirt1: silent mating type information regulation 2 homolog 1
TAA: thoracic aortic aneurysm
VVG: Verhoeff van Gieson
VSMC: vascular smooth muscle cells.
