# Supplementary figures and images for: Profiling of Histone Modifications Reveals Epigenomic Dynamics During Abdominal Aortic Aneurysm Formation in Mouse Models
Source: Front Cardiovasc Med. 2020 Oct 30;7:595011. doi: 10.3389/fcvm.2020.595011 (PMC7662126; doi:10.3389/fcvm.2020.595011)

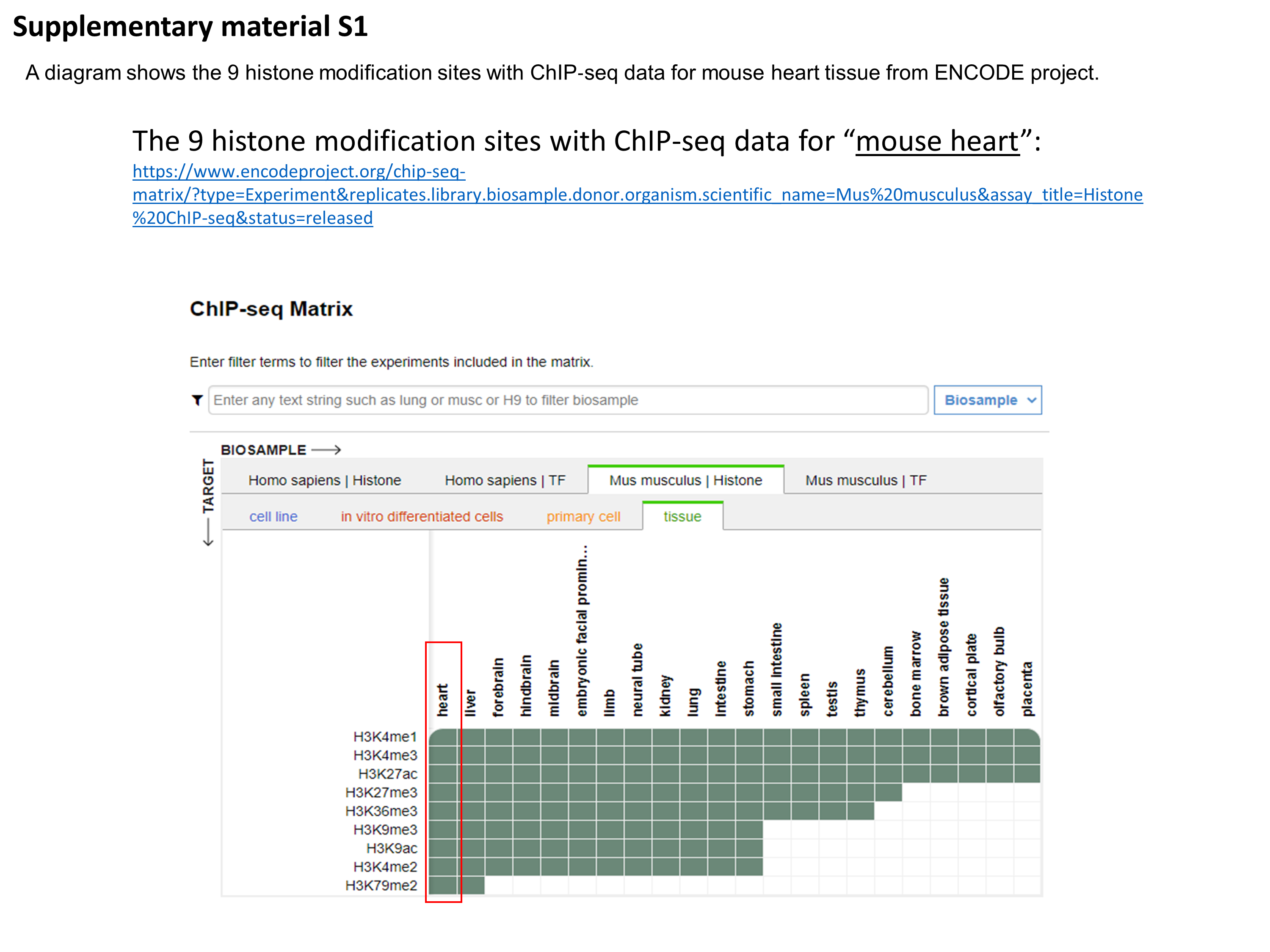

Supplement: Supplementary file 1 [file Image_1.TIF]

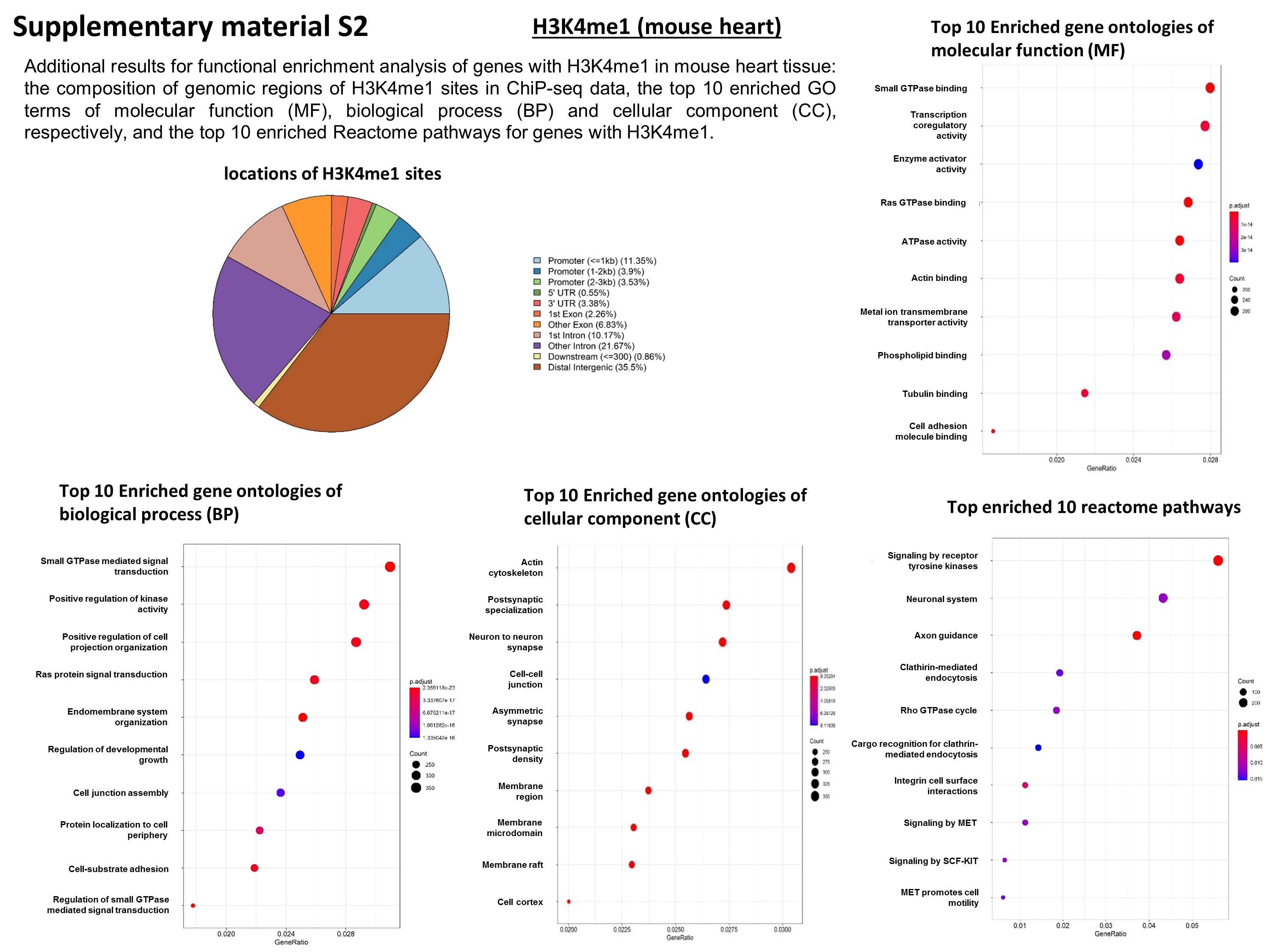

Supplement: Supplementary file 2 [file Image_2.TIF]

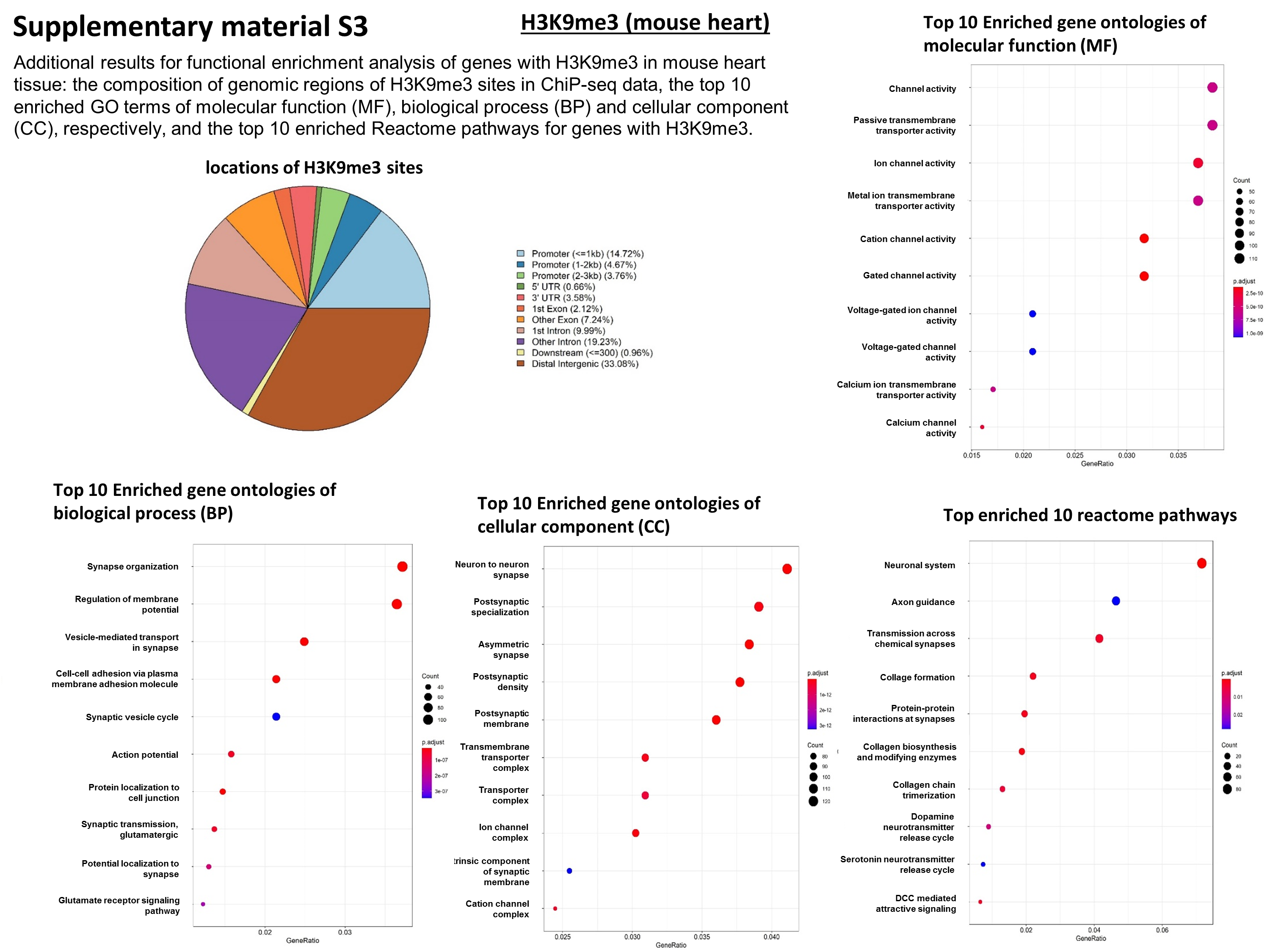

Supplement: Supplementary file 3 [file Image_3.TIF]

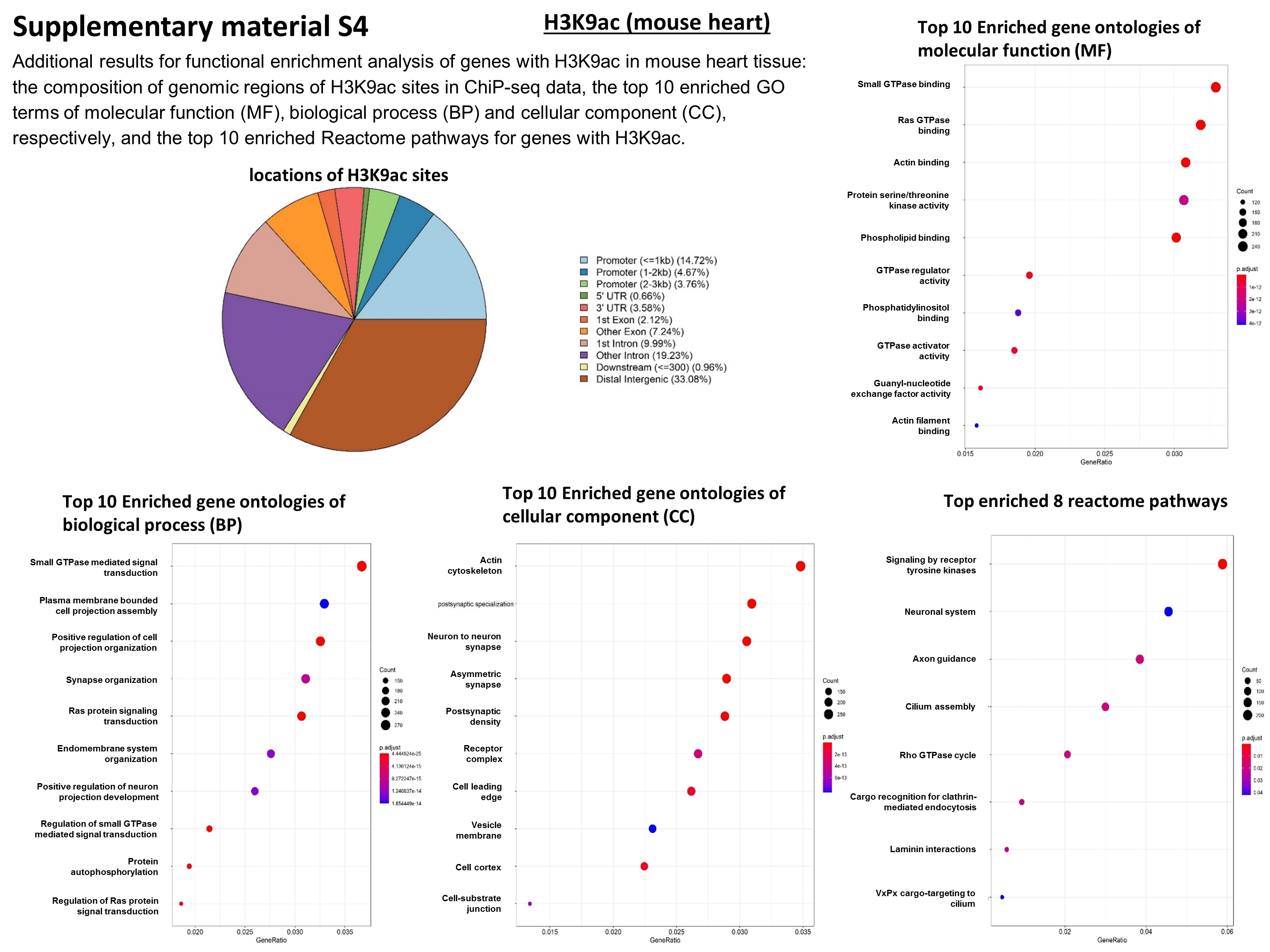

Supplement: Supplementary file 4 [file Image_4.TIF]

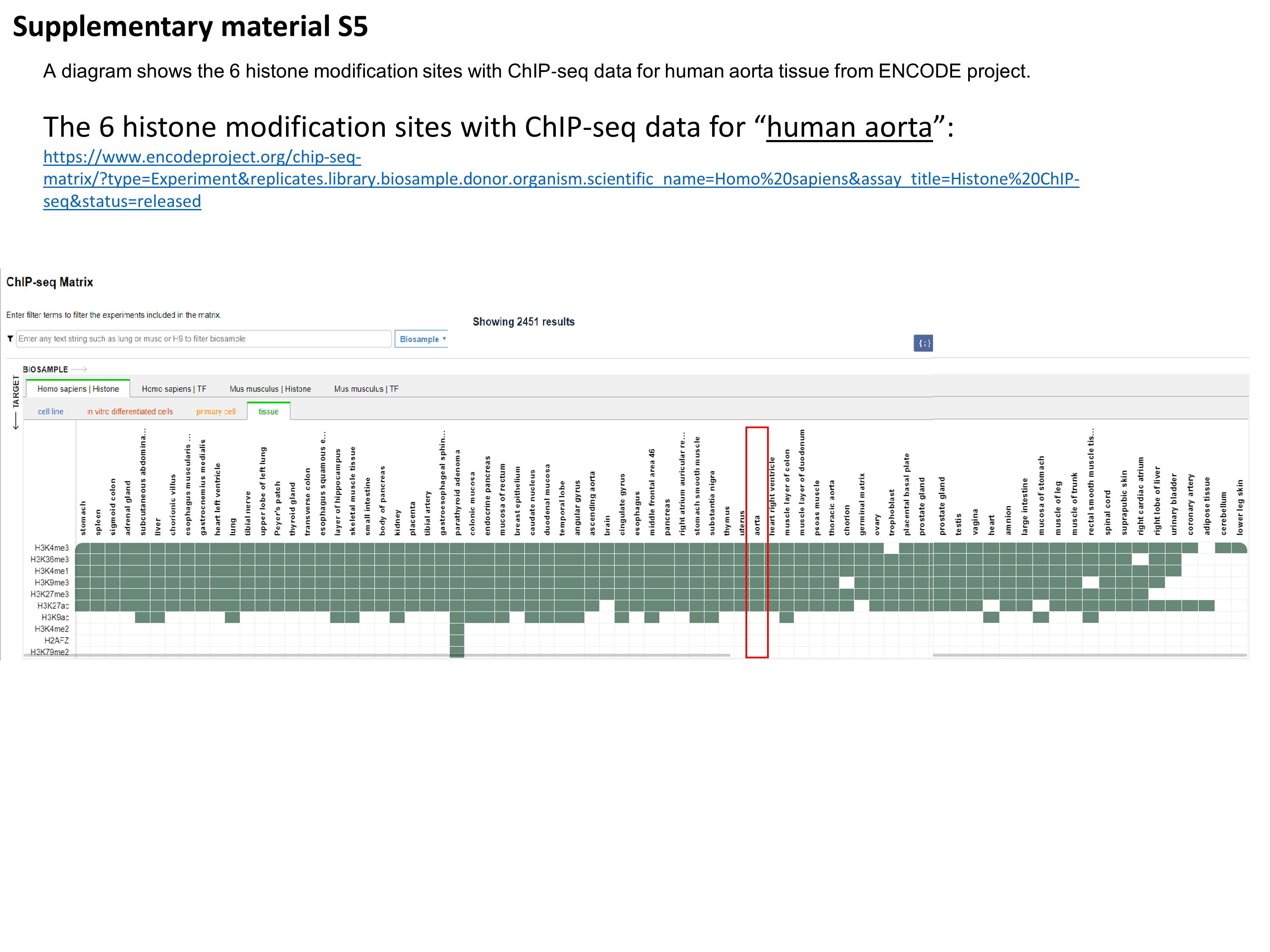

Supplement: Supplementary file 5 [file Image_5.TIF]

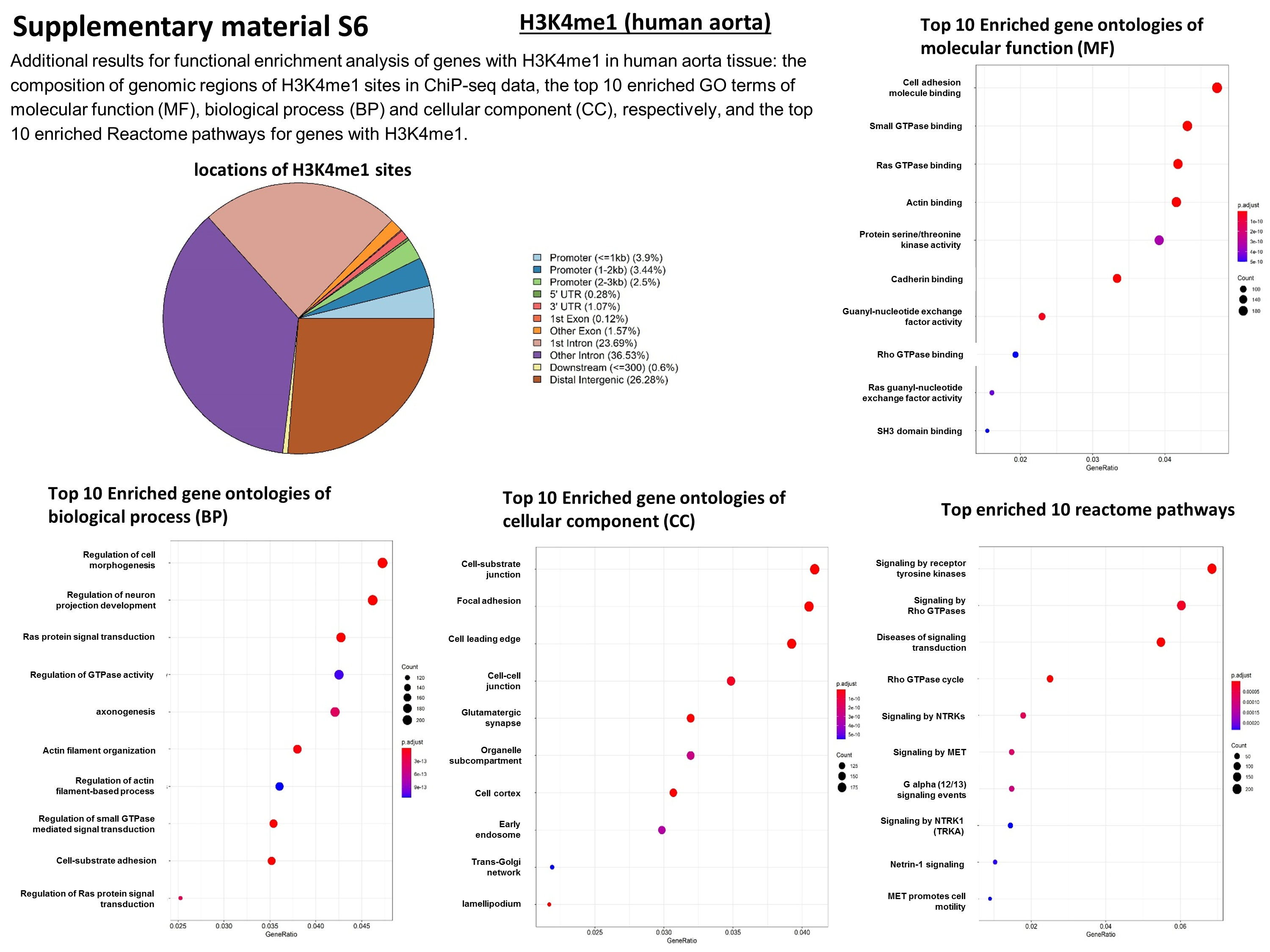

Supplement: Supplementary file 6 [file Image_6.TIF]

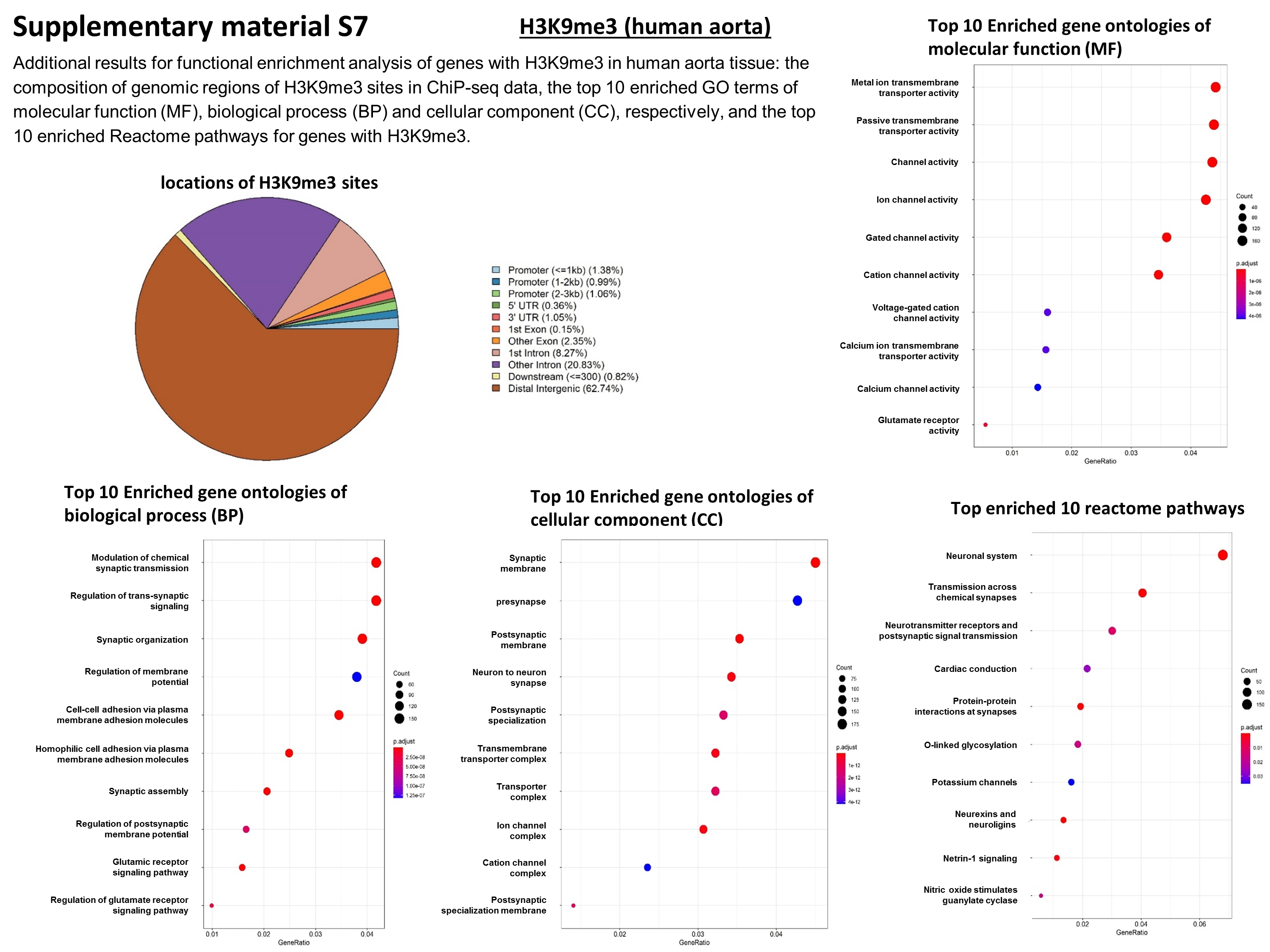

Supplement: Supplementary file 7 [file Image_7.TIF]

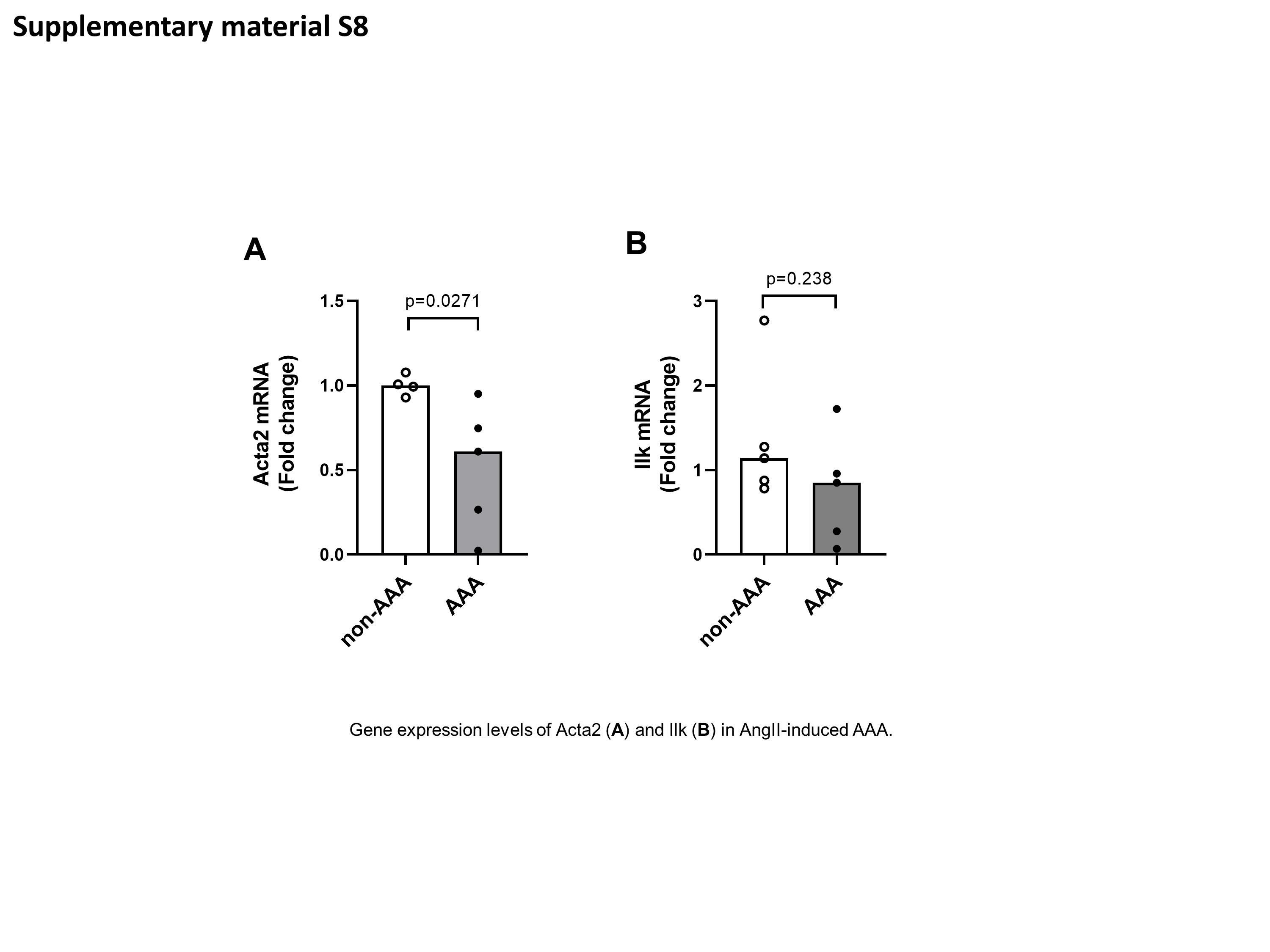

Supplement: Supplementary file 8 [file Image_8.TIF]
